# Selective small molecule targeting of KDM4 as a therapeutic strategy to reduce proliferation of acute myeloid leukaemia

**DOI:** 10.1111/bjh.70351

**Published:** 2026-02-01

**Authors:** Laura Monaghan, Roderick P. Bunschoten, Joana Bittencourt‐Silvestre, Taeju Park, Susan Gannon, Petrisor Alin Pirvan, Alex Hoose, Helen Wheadon, Robert M. J. Liskamp, Xu Huang, Heather G. Jørgensen

**Affiliations:** ^1^ Paul O'Gorman Leukaemia Research Centre, School of Cancer Sciences University of Glasgow Glasgow UK; ^2^ Medicinal Chemistry Department, School of Chemistry University of Glasgow Glasgow UK; ^3^ Cardiovascular Research Institute Maastricht (CARIM) Maastricht The Netherlands; ^4^ Johnson & Johnson Innovation Shanghai China

**Keywords:** AML, epigenetics, histone demethylase, KDM4, PARP‐1

## Abstract

Acute myeloid leukaemia (AML) is an aggressive disease with poor survival and high relapse rates. Coupled with the complex mutational burden observed, there is an unmet clinical need for more targeted therapies. Epigenetic therapies have shown promise both as monotherapy and in combination strategies and specifically histone lysine demethylase, KDM4A (Lysine demethylase 4), plays a role in the maintenance of AML, with its short hairpin (shRNA) knockdown sufficient to target leukaemia cells while sparing normal haemopoietic cells. In this study, we utilised a novel KDM4 inhibitor based on the structure of IOX‐1, the most characterised inhibitor of the 2‐oxygenase enzymes to which the KDM4 family belong, to investigate further the role of KDM4A in AML. Our compound induced AML cell death with cell cycle arrest, failure of colony formation and transcriptomic changes in metabolism, transcription control and response to stress. With known roles for KDM4A family members in deoxyribonucleic acid (DNA) damage response repair pathways, inhibition of KDM4A increased accrual of double strand DNA breaks. Hence, we demonstrated KDM4i sensitisation of leukaemia cells to inhibitors of DNA damage pathways such as poly‐ADP ribose polymerase (PARP) inhibitor, olaparib, suggesting future clinical evaluation of KDM4A and other key components in DNA damage/response signalling pathways as potential therapeutic vulnerabilities in AML.

## INTRODUCTION

Mutations that drive acute myeloid leukaemia (AML) resulting in enhanced self‐renewal ability and consequent accumulation of immature myeloid cells occur in several different functional groups including transcription factor regulators, signalling proteins^1^ and splicing factors.^1^ The heterogeneity of the disease at molecular, morphological and clinical levels poses a therapeutic challenge with varying responses to treatment, resistance and relapse. Recognition of the influence of epigenetics, specifically the enzymes that control modifications, on AML development and maintenance offers the opportunity to explore the potential for pharmacological targeting.^2^, ^3^, ^4^, ^5^

It has been established that the KDM4 family of histone demethylases, specifically Lysine demethylase 4 (KDM4A), has a role in AML and other cancers.^6^, ^7^ More specifically, KDM4A was shown to be required for the initiation and maintenance of AML,^8^, ^9^ with short haripin (shRNA) depletion in human cells selectively inducing leukaemic cell apoptosis, sparing the normal bone marrow cells.^8^, ^10^, ^11^

Belonging to the Jumonji (Jmj) domain containing demethylases (JMJD), the JMJD2 or KDM4 family of histone demethylases has many roles postulated in cancer progression alongside normal cellular development.^12^, ^13^ Functioning in an Fe^+^ and α‐ketoglutarate (KG)‐dependent manner, KDM4A, catalyses the demethylation of H3K9me3 and H3K36me3, controlling gene expression, apoptosis and transcription activation. KDM4A overexpression in cancer is associated with genomic instability resulting in accelerated replication due to an increase in chromatin accessibility. Conversely, loss of KDM4A also has roles in centrosome maintenance in which its loss in normal development may result in widespread genomic changes.^14^, ^15^, ^16^ Alternative roles for KDM4A, both dependent or independent of its demethylase activity, have been postulated, such as in cell cycle progression and interaction with translation machinery.^17^, ^18^ Notably, global H3K9me3 hypomethylation was prevalent in primary AML patient blasts compared with normal CD34+ haemopoietic stem and progenitor cells (HSPCs) with the potential to stratify AML patient outcome solely on differential expression owing to changes in H3K9me3 levels.^19^

The most successful inhibitor of the KDM4 demethylases to date was identified in 2010.^20^ Designed as a pan‐KDM inhibitor, IOX‐1 (5‐carboxyl‐8‐hydroquinoline), has a binding affinity for KDM4A and other members of the KDM4 family but low cell permeability. IOX‐1 also inhibits other 2‐oxoglutarate (OG)‐dependent oxygenases, such as hypoxia inducible factor‐1alpha (HIF‐1α), which is suggested to be tumour suppressor in certain types of cancers. Further derivatives of IOX‐1 have shown improved specificity for targets such as HIF‐1α with success in preclinical models of AML^21^ and proteolysis targeting chimera (PROTAC) molecules targeting other KDM4 family members are being investigated in a range of cancer types.^22^

We developed a series of novel KDM4 family small molecule inhibitors based on IOX‐1 with the intention to improve upon cell permeability and specifically allow further investigation of KDM4A and its roles and interactions in AML.

## MATERIALS AND METHODS

### Cell culture

Primary samples were obtained from the Paul O'Gorman Leukaemia Research Centre research tissue bank (Research Ethics Committee [REC] 4 approval; 20/WS/0066) or from collaboration with Professor Sandra Marmiroli at UNIMORE, Modena, Italy, with informed consent in accordance with the Declaration of Helsinki.

### Drug preparation

Small molecule inhibitors of KDM4 (KDM4i) were synthesised within Liskamp laboratory at the University of Glasgow Chemistry department. Each was reconstituted in dimethylsulphoxide (DMSO); 0.1% (v/v) DMSO was used as a vehicle control unless otherwise specified. The PARP inhibitors (PARPi)—olaparib (AZD‐2281), veliparib (ABT‐888) and talazoparib (BMN‐673)—were purchased from SelleckChem (Houston, USA).

### RNA extraction and library preparation

Total RNA was extracted from <10^5^ cells treated for 36 h with varying conditions using the Qiagen RNeasy Micro kit with high‐quality RNA (500 ng) utilised from triplicate repeat treatments to create a complete RNA Library using the Illumina TruSeq Stranded mRNA. Samples were pooled to create a single library and sequenced at University of Edinburgh Genomics Centre on the Illumina NovaSeq system generating 50 paired‐end read data. Data files are available in the Gene Expression Omnibus (GEO): GSE125376, GSE164437.

## RESULTS

### Pharmacological inhibition of the KDM4 family reduced *MLL* ‐rearranged AML cell line numbers to different degrees

**FIGURE 1:**
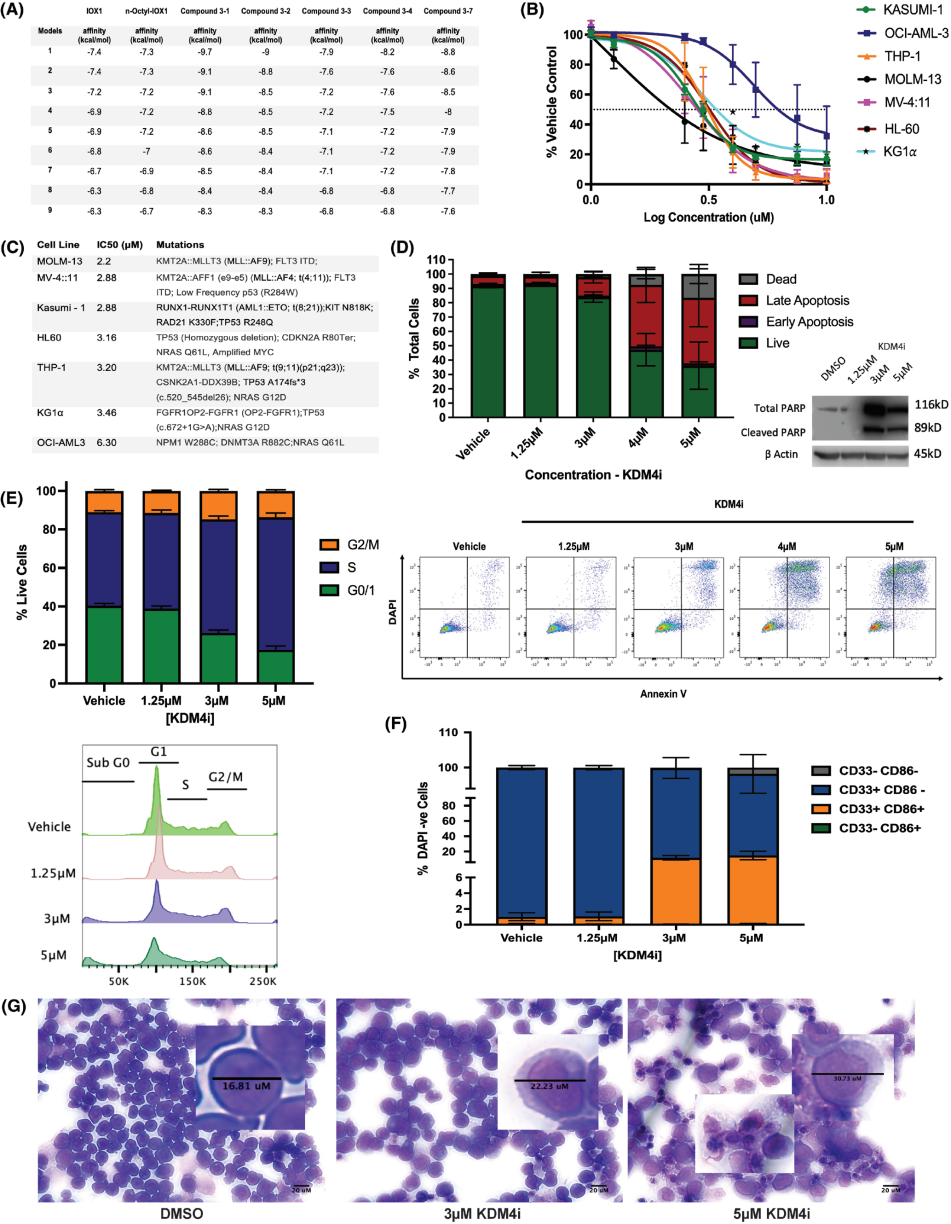
*Pharmacological inhibition of KDM4 reduces the* viability *of different acute myeloid leukaemia (AML) cell* lines with cell cycle arrest and loss of cellular structure. (A) Binding affinities of our novel KDM4 inhibitors in comparison with IOX‐1 and n‐octyl ester derivative through molecular docking simulations whereby greater negative affinity (kcal/mol) is indicative of greater perceived binding. (B) A range of AML cell lines with varying molecular subtypes were treated for 48 h with novel KDM4i (Compound 3‐7) in monotherapy. IC_50_ was determined followed by a 4‐h incubation with resazurin, and the percentage change was calculated relevant to the vehicle (*n* = 3). (C) Table representing cell lines ranked by IC_50_/EC_50_ values and highlighting the important mutations of each cell line to allow for further analysis of differences in response, *n* = 3, biological replicates each with technical triplicates. THP‐1 cells were treated for 48 h and different analyses were carried out. (D) Apoptosis phenotype was assessed by Annexin V/DAPI flow cytometry and accumulation of cleaved PARP with increasing concentration of KDM4i was calculated. % of cells in each stage of apoptosis cycle. Stages of apoptosis are gated as a percentage of total cell, with representative dot plot images (*n* = 3, biological replicates) alongside representative western blot image of accumulation of cleaved PARP (*n* = 3, biological replicates). (E) THP‐1 cells were fixed and stained by PI/RNase A solution for DNA staining; the cells were acquired immediately through flow cytometry. Representative histogram of stages of cell cycle assessed by curve fitting the cell cycle with the percentage of cells in each cycle phase of live cells (excluding SubG0) showed an increase in S phase with increasing concentration. Bars show mean ± SEM, *n* = 3, biological replicates. (F) Gain of CD86, a dendritic cell surface maturation marker by THP‐1 cells upon treatment with KDM4i monotherapy for 48 h, followed by flow cytometry analysis (*n* = 3, biological replicates). (G) May–Grünwald–Giemsa staining of THP‐1 cells treated for 48 h with Compound 3‐7 showing changes in morphology and cell death. Dimethylsulphoxide (DMSO)‐treated cells have a small size with intact structures and large nuclear to cytoplasmic ratios. Low concentration of Compound 3‐7 shows an increase in overall cell size with a reduction in nuclear to cytoplasmic ratio. High concentration of Compound 3‐7 shows further increase in the size of cells with loss of cell structure indicative of cell death.

Seven AML cell lines representative of varying AML subtypes were selected to establish the effect of our KDM4 family pharmacological inhibitor known as Compound 3‐7 (‘KDM4i’). Binding specificity superiority over IOX‐1 and its derivative *n*‐octyl‐IOX‐1 was established using nine varying docking simulations to predict the binding affinity (kcal/mol) towards KDM4A. Of our novel compounds with higher binding affinity than their common antecedants, that is, Compounds 3‐1, 3‐2 and 3‐7 (Figure 1A), each was trialled for cell permeability with Compound 3‐7 selected as best for affinity, permeability and potency (Figure S1A). Following 48‐h KDM4i monotherapy treatment, non‐linear regression modelling identified differences in the responses with respect to 50% inhibitory concentration (IC_50_) for live cell numbers measured by resazurin (Figure 1B). Of the seven cell lines tested, some were highly sensitive (MOLM‐13; IC_50_ 2.2 μM); one responded poorly (OCI‐AML3; IC_50_ 6.3 μM); the majority had intermediate sensitivity (THP‐1, HL60, MV: 4–11 and KG‐1α) with IC_50_ ranging from 2.9 to 3.5 μM (Figure 1C). Importantly for the potential of combination therapies, some of the cell lines found to be of intermediate sensitivity to KDM4i have known p53 mutations, potentially rendering them p53 null (e.g. THP‐1) or with reduced p53 function that impairs their ability to repair DNA damage.^23^

### Compound 3‐7 (KDM4i) induces an apoptotic phenotype with cell cycle arrest and loss of cellular structure

THP‐1 cell line (Compound 3‐7 IC_50_ is 3.2 μM) was selected for further study. As this study aimed to understand the response of mixed‐lineage leukemia (*MLL*) rearranged AML to epigenetic inhibition of KDM4A, owing to their *MLL‐AF9* translocation (*KT2A::MLLT3*; t(9:11)(q21;q23)) and the availability of our published dataset following genetic knockdowns (KD),^8^ THP‐1 cells were chosen for the majority of the experimental work. Confirmation of results observed was carried out in MV4‐11 cells with an alternative *MLL::AF4* translocation (Figure S2). A concentration‐dependent increase in apoptosis was evident (Figure 1D) in keeping with the negative sigmoid concentration–effect curve. Accumulation of cleaved PARP was observed by western blotting confirming initiation of cell death with KDM4 inhibition by KDM4i monotherapy.

Accumulation of THP‐1 cells in the sub‐G0 phase of the cell cycle where DNA content is <2n (Figure 1E) was indicative of cell death and DNA fragmentation. Accumulation of cells in the S‐phase together with a loss of cells in G1 phase indicated S‐phase cell cycle arrest in response to KDM4i. With visualisation of the cellular morphology by May–Grünwald–Giemsa staining (Figure 1G), overall enlargement of cells alongside a reduction in nuclear to cytoplasmic ratio were indicative of differentiation to the myeloid lineage. Differentiation could be confirmed with the accumulation of a population of CD86‐positive cells, a known marker of lineage commitment (Figure 1F).^24^, ^25^, ^26^

### Differential gene expression with KDM4i in comparison with lentiviral KDM4A KD or IOX‐1 treatment

**FIGURE 2:**
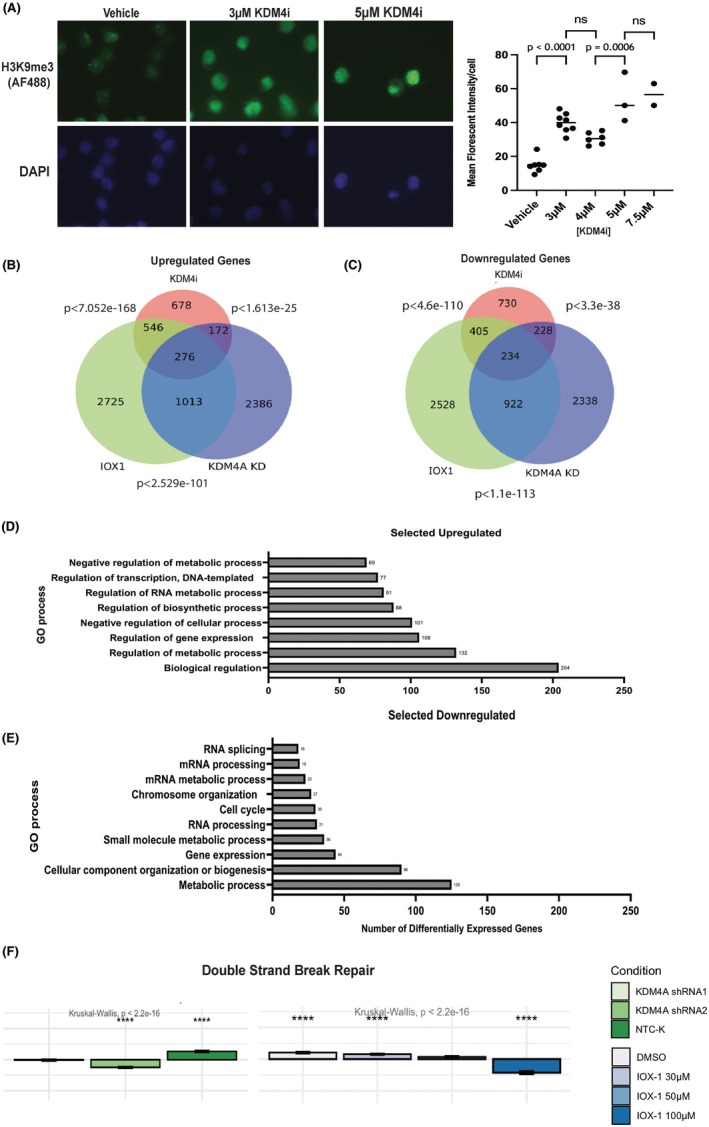
*Gene expression changes following THP‐1 treatment with* KDM4i, *KDM4A shRNA KD and IOX‐1* indicate cellular metabolsim changes and DNA repair pathways in mechanism. (A) THP‐1 cells were treated for 72 h with Compound 3‐7, the cells were then fixed and stained for H3K9me3 (Alexa Fluor 488—Green) and imaged using the AxioVision M1 fluorescence microscope. 4′,6‐diamidino‐2‐phenylindole (DAPI) was used as a nuclear counterstain. Images taken at 100× magnification. Representative images of *n* = 1 biological replicate with reproducible results confirmed by multiple fixations. Quantification of signal carried out by image J Fiji. (B, C) RNA Sequencing of KDM4A KD by shRNA, treatment by IOX‐1 for 48 h and treatment with Compound 3‐7 for 36 h was followed by differential expression analysis. Venn diagram of common and unique (B) upregulated and (C) downregulated genes between the conditions. *p* Values calculated by hypergeometric testing. (D, E) RPA of 276 genes common to all three treatments identified (D) Selected significantly upregulated pathways ordered by enriched genes, including metabolic process and transcriptional signalling. (E) Selected significantly downregulated pathways ordered by number of enriched genes, including metabolic processes, cell cycle and chromatin organisation. (F) KDM4A shRNA KD and IOX‐1 treatments reduce the gene expression of genes involved in double‐strand break repair. A significant reduction in double‐strand break repair was observed by calculation of a *Z* score. For each gene within a gene set, the average gene expression of all genes is subtracted and this value divided by the standard deviation to assign a score for the changes observed in the total gene set. **** *p* < 0.0001. Values represent the mean of three replicates with statistical analysis by one‐way ANOVA with multiple comparison correction. NTC‐k represents a non‐targeting control shRNA construct as a negative control.

Immunofluorescence microscopy indicating accumulation of H3K9me3 upon KDM4i treatment correlated with the expected reduction in KDM4A demethylase activity following drug treatment (Figure 2A; Figure S1B).

To further clarify the specific targeting of KDM4A and delineate the specific phenotype of KDM4A reduction, RNA seq previously carried out after either shRNA KD of *KDM4A* in THP‐1 cells (GEO: GSE125376)^8^, ^9^ or IOX‐1 treatment, was analysed alongside new RNA seq transcriptomic data generated in this study. Treatment of THP‐1 with KDM4i (at its IC_50_ for 36 h) showed a smaller number of upregulated or downregulated genes (678 up; 730 down) in comparison with *KDM4A* shRNA KD or IOX‐1 (>2300 up or down unique differentially expressed genes (DEGs)) (Figure 2B,C). When specific gene ontology (GO) terms were considered, commonly upregulated pathways included response to stress and hypoxic starvation alongside regulation of metabolic processes (Figure 2D). Downregulated pathways (Figure 2E) included DNA replication and unwinding capacity, alongside RNA processing and mRNA splicing, extended GO term analysis in Figure S3. Importantly for consideration of potential combination therapies, there was a significant downregulation of DNA repair pathways, with KDM4 loss by shRNA KD or by IOX‐1 treatment (Figure 2F).

### KDM4i sensitises AML cell lines to PARP inhibition by olaparib

**FIGURE 3:**
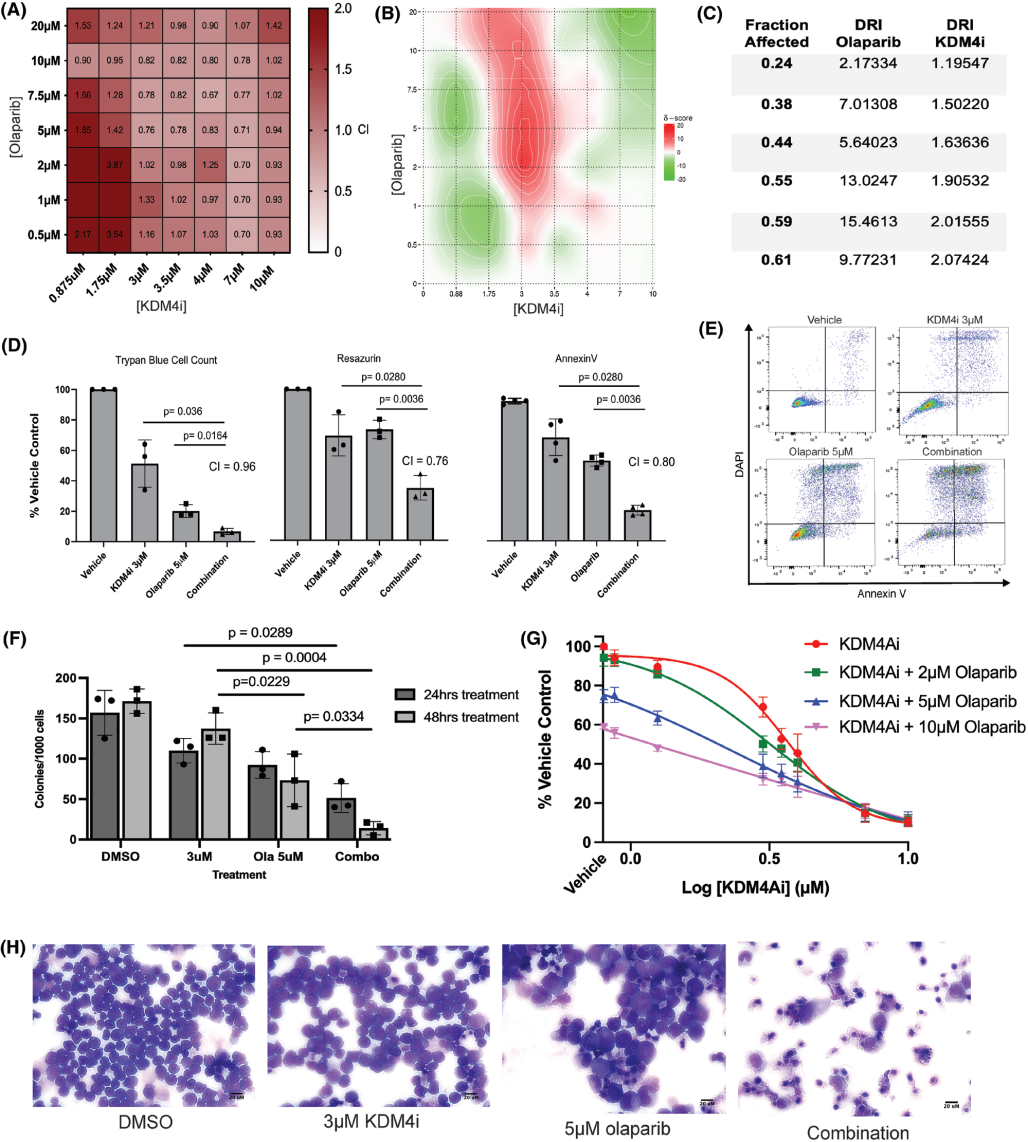
*KDM4 synergises with olaparib in THP‐1 cells to induce increased cell death.* Varying concentrations around the IC_50_ of both compounds were in combination at time 0 h in THP‐1 cells. Inhibition effect was determined using resazurin and the combination index (CI) calculated by CompuSyn. CI <1 is synergistic, CI ~1 is additive and CI >1 is antagonistic. (A) Heat map of representative CI values was generated using GraphPad (*n* = 3). (B) Combination analysis of the same inhibition data was carried out using the Bliss Independence method using Synergy Finder and confirmed the synergistic effect of the combination treatment. The synergy scores are represented on a heat map with hotspots of synergy (scores below −10) represented by red colouring, and spots of antagonism (above 10) in green, white areas (−10 to 10) represent the two drugs being additive at those concentrations. (C) Dose reduction index of both drugs when used in combination calculated using CompuSyn representing the perceived reduction in the dose of each compound which is sufficient in combination to achieve the same effect as the monotherapy. (D) Confirmation of synergy by trypan blue cell counts (*n* = 3), resazurin (*n* = 3) and Annexin V/DAPI flow cytometry (*n* = 3) with representative images (E) and (D) Independent calculation of the bliss synergy score using the bliss equation. (F) Colony‐forming potential of cells following treatment for 24 or 48 h before drug was removed and cells resuspended in semisolid media. Colony numbers per 1000 plated for each condition after treatment for 24/48 h, respectively, with representative images of colonies produced by each condition after 7 days (*n* = 3). (G) IC_50_ curves of combinations of varying concentrations of KDM4i with a constant olaparib concentration (*n* = 3). (H) Cytospin preparations of cells treated for 48 h with monotherapy or combination showing cell death and loss of cellular structure.

Considering THP‐1 cells, a synergistic effect of combining KDM4i with the PARPi, olaparib, could be demonstrated by calculation of combination index (CI)^27^ (Figure 3A). This observation was not specific for *MLL* rearrangements with synergy also confirmed in other cell lines representing varying AML subtypes such as OCI ALM3, Kasumi 1 and KG1alpha (Figure S4).

The synergy of the two compounds was confirmed using the Bliss Independence method^28^ allowing for a dose reduction index (DRI) to be calculated (Figure 3B,C). The DRI (Figure 3C) identifies how many fold the dose of each drug in a synergistic combination can be reduced to elicit the same effect as monotherapies considered alone (Figure S4). To elicit an IC_50_ reduction in cell viability, a 13‐fold dose reduction of olaparib and a 1.9‐fold reduction in KDM4i would be equivalent.

The synergistic combination of olaparib (5 μM) and KDM4i (3 μM) was independently confirmed by trypan blue cell counts, resazurin and flow cytometry for apoptosis (Figure 3D,E). A significant reduction was seen in cellular metabolism and cell count with an increase in apoptosis with the combination treatment (24/48 h) in comparison with either monotherapy (*p* < 0.036), accompanied by a loss in the capacity of the cells to form colonies with 24‐ or 48‐h treatment followed by removal of the drug and semi‐solid culture for a subsequent 7 days (Figure 3F). This could be further confirmed by considering the IC_50_ dose–response curves (Figure 3G), and cytospin for the combination which shows a complete loss of structure (Figure 3H).

### Combination treatment reduces proliferation and is maintained following drug removal with increased damage accrual

**FIGURE 4:**
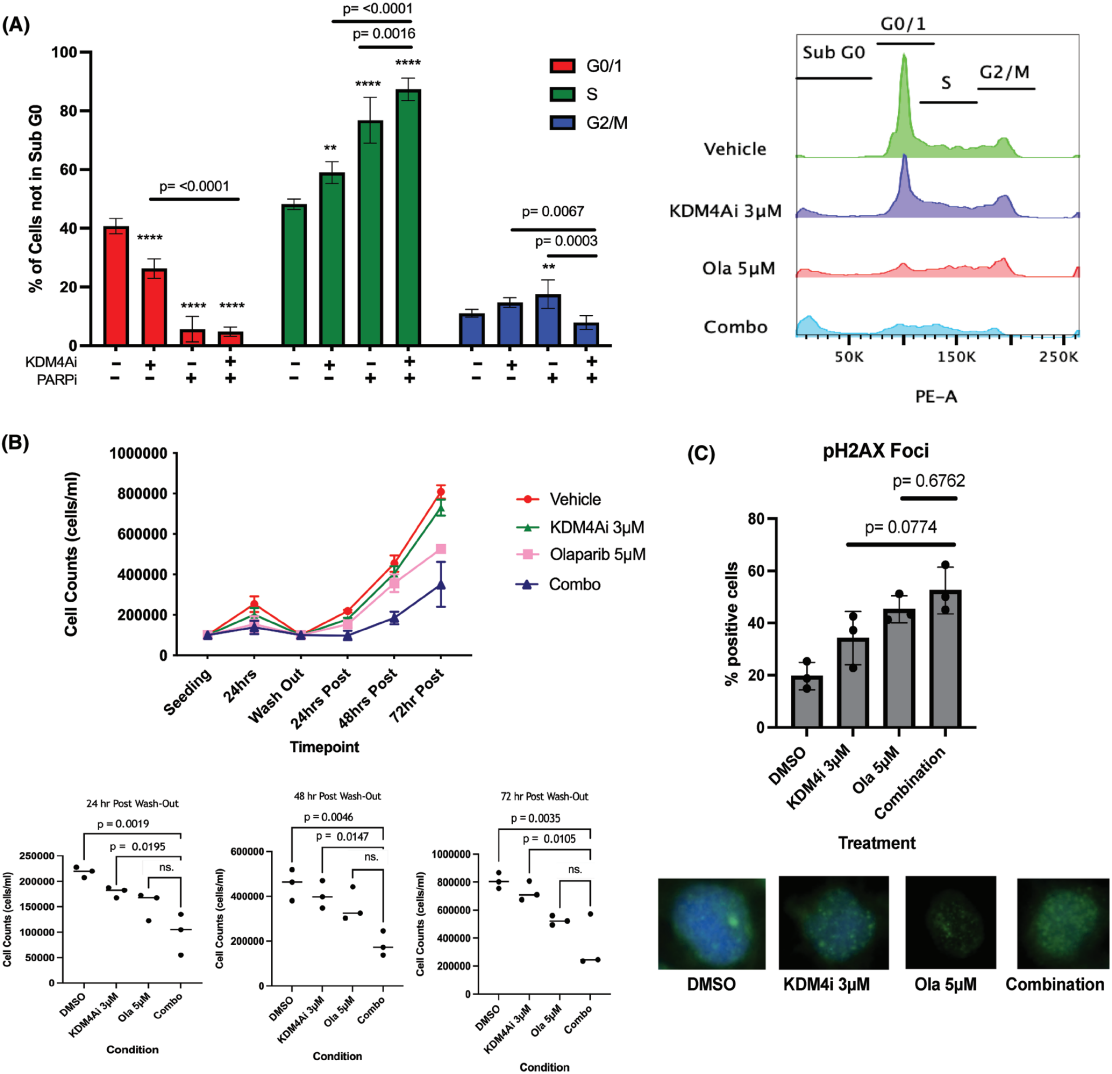
*Combination treatment induces a cell cycle arrest with sustained effect and increased accrual of DNA damage in THP‐1 cells.* THP‐1 cells were treated alone or in combination with Compound 3‐7 for 48 h before cells were fixed and analysed by PI/RNAse A solution for staining of DNA content, each of the cell cycle phases were identified before live cells (minus subG0) were further analysed for cell cycle stage (A) Bars represent mean ± SD, *n* = 3, biological replicates, statistical analysis by one‐way ANOVA with multiple comparison. Asterisks represent significant difference from vehicle control, ** *p* < 0.01 **** *p* < 0.0001, *p*‐values represent significance between treatments. Alongside profiles of each sample cell cycle stages. (B) Delayed growth of cells with 24‐h treatment followed by 72‐h observation. Cells were treated for 24 h with Compound 3‐7 and olaparib alone or in combination before the drug was washed out and the cell growth monitored for further 72 h by trypan blue cell counts shown as cells/mL. Overall growth of cells for observation period, dots show mean ± SEM, *n* = 3, biological replicates each as a mean of technical duplicates. Individual analysis of 24, 48 and 72 h post wash‐out cell growth with statistical analysis by one‐way ANOVA with multiple comparison. (C) Immunofluorescence analysis of THP‐1 cells treated for 48 h. with Compound 3‐7 and olaparib alone or in combination. Positive cells were assessed as having >5 foci and calculated as a percentage of total cells in the field of view. Bars represent mean ± SD, statistical analysis by one‐way ANOVA with multiple comparison *n* = 3 biological replicates.

**FIGURE 5:**
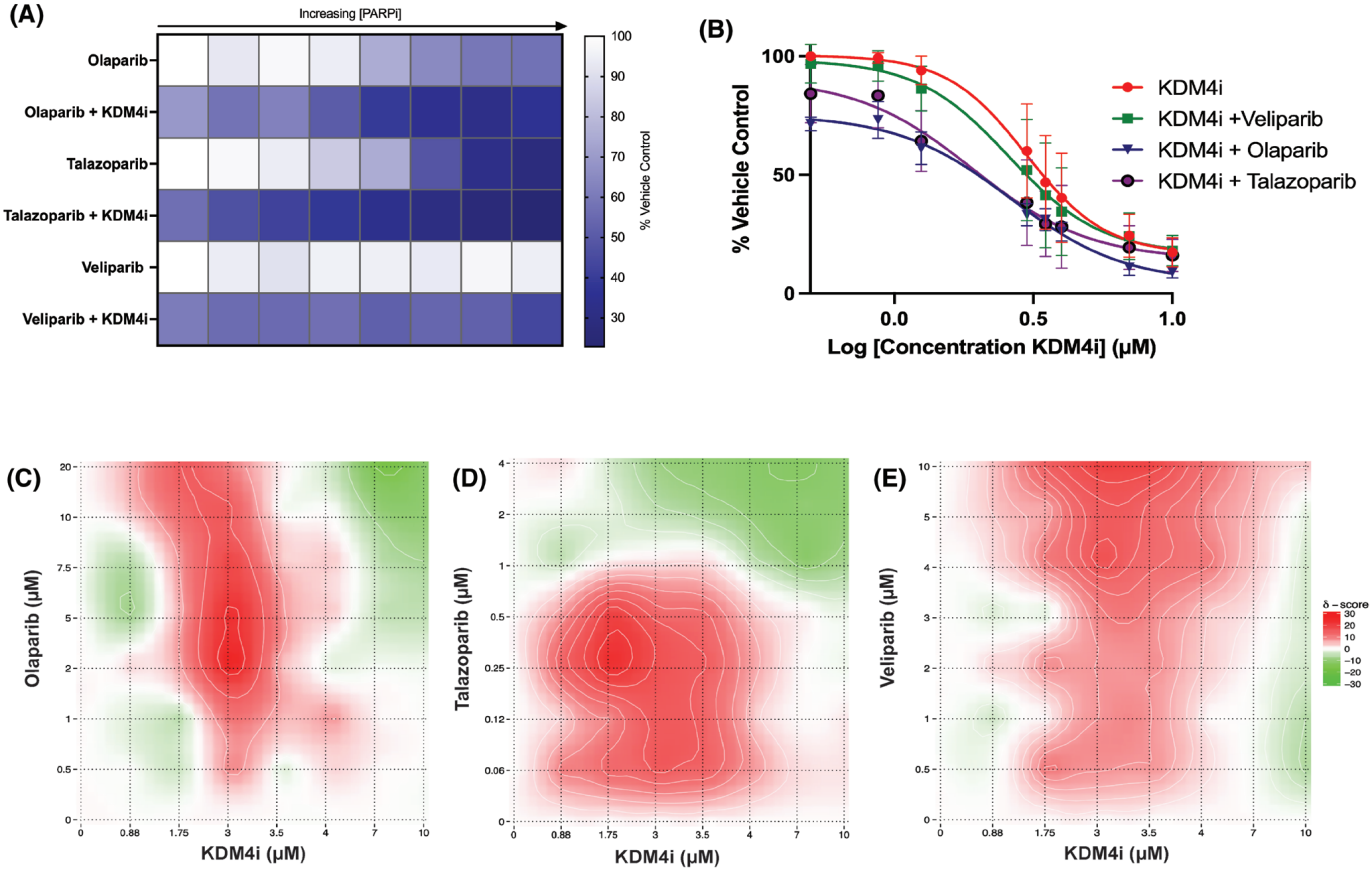
*Degree and concentration of synergism is influenced by the PARP trapping potential of different PARP inhibitors*, increased expected PARP trapping increases effectivness of combination. (A) IC_50_ curve of KDM4i treatment alone for 48 h in THP‐1 cells on comparison with a fixed concentration of each PARPi combined with a varying range of KDM4i concentrations. (B) When each PARPi is used in turn in combination with Compound 3‐7 in THP‐1 cells, the varying effects on cell death can be observed with KDM4i combined with talazoparib showing the most synergistic and effective combination, *n* = 3, biological replicates each as a mean of technical triplicates. Dots represent mean ± SEM. (C–E) Bliss independence analysis of synergy using Synergy Finder following 48‐h treatment, and normalised resazurin fluorescence with each PARPi in turn (C) olaparib, (D) talazoparib, (E) veliparib. The synergy scores are represented on a heat map with hotspots of synergy (scores below −10) represented by red colour, spots of antagonism (above 10) in green and white areas (−10 to 10) represent the two drugs being additive at those concentrations.

KDM4i alone arrested THP‐1 cells in S/G2M phases with a decreased ratio of cycling to non‐cycling cells, which was also observed with olaparib monotherapy (Figure 5A). Despite a similar ratio of cycling to non‐cycling cells to PARPi monotherapy, the combination treatment caused a significantly increased S‐phase arrest with respect to either therapy alone (<0.001 and 0.0016 respectively; Figure 4A).

At low concentrations of KDM4i monotherapy (1.25–3 μM), the cells could proliferate normally following drug removal by washout (Figure 4B; Figure S5). Cells treated with olaparib recovered their growth pattern after the drug was removed which, although slower than the vehicle, trended towards normality. The cell number with the combination treatment remained static without growth for the first 24 h, with a failure to expand to the same extent in the subsequent observation time after drug removal (Figure 4B). DNA damage was analysed by the accumulation of ƔH2Ax by immunofluorescence following combination treatment of KDM4i with olaparib for 24 h (Figure 4C). Olaparib alone significantly increased foci with more foci observed with the combination treatment.

### Synergistic potential of KDM4 inhibition with PARPi is influenced by PARP trapping

**FIGURE 6:**
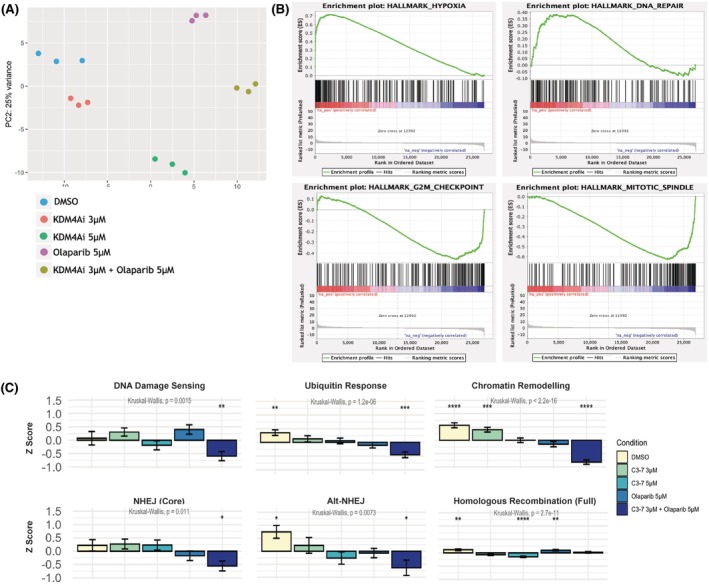
*Combination increases cell death through impaired cellular response to stress and DNA damage with KDM4i sensitising cells to the effect of PARPi olaparib.* THP‐1 cells were treated with Compound 3‐7 and olaparib alone or in combination for 36 h and samples prepared for RNA sequencing. (A) Principal component analysis identified variance between the samples, following batch correction the samples clustered in replicates according to variance. (B) GSEA of differentially expressed genes identifies an upregulation of cellular responses to stress in the combination treatment when the effect of the monotherapies has been removed to leave only synergistic genes altered in the combination alongside a downregulation of gene sets involved in cell cycle control. (C) Average *Z* score calculated for all components of pathways involved with or associated to DNA damage and repair *n* = 3 statistical analysis by Kruskal–Wallis one‐way ANOVA, * represents significant difference in each group on comparison with the mean of all values ** *p* < 0.01, *** *p* < 0.001 **** *p* < 0.0001.

PARPi can be characterised by their potential to trap PARP on damaged DNA, resulting in an increased time of association between PARP1 and sites of damage; if PARP1 is not appropriately removed to allow binding of DNA damage repair factors, damage persists and can result in cell death.^29^, ^30^ With this knowledge, we utilised three PARPi, with varying degrees of PARP trapping potential^30^ to observe differences in monotherapy and combination (Figure 5A). Olaparib has an intermediate ‘PARP trapping’ potential when compared to veliparib (ABT888) (the lowest) and talazoparib (BMN673) (the highest). There is no significant advantage to combining KDM4i with veliparib as the combination curve mirrors that of KDM4i as a single inhibitor (Figure 5B). With the other compounds, it is possible to observe a left shift in the curve confirming the cooperative effect as confirmed by Bliss. In comparison with olaparib (Figure 5A), talazoparib alone has the greatest potency as monotherapy against AML cell lines (Figure 5A), and in combination with KDM4i at concentrations around its IC_50_ (1.75 μM), lower than that observed with olaparib, there is a largely synergistic effect (Figure 5B). When the three PARPi are compared (Figure 5C–E), the differences in where synergy lies with the combinations become more apparent; reduction of KDM4 activity modifies chromatin structure thus trapping PARP on DNA hence reducing the cell's capacity to recognise further accrual of damage (Figure 6C). The degree to which PARP trapping influences the cytotoxic mechanism of the inhibitors and whether PARP trapping is cell context dependent is subject to further investigation.

### KDM4 inhibition sensitises cells to PARPi through the improper stress response and inflammatory processes and combination reduces gene expression of several DNA damage‐associated pathways

RNA sequencing carried out after 36 h treatment with either monotherapy or combination showed distinct transcriptomic profiles (Figure 6A). Principal component 1 (PC1) accounts for 53% of variance, following batch correction for the three replicate samples; PC1 clusters DMSO with 3 μM KDM4i to the left axis, 5 μM KDM4i sits in the middle, with olaparib and combination treatment clustering to the right. PARPi accounts for greater variance than changes observed with KDM4A, leading to the hypothesis that KDM4i sensitises the cells to PARPi, olaparib.

Gene set enrichment analysis (GSEA) analysis of combination treatment in comparison with either monotherapy alone identified a significant change in several cellular stress responses (Figure 6B; Figure S6). The most enriched pathways by genes upregulated by the combination were hypoxia (normalised enrichment score (NES): 2.67, *p* < 0.001) and cellular stress. Overrepresentation of mTOR signalling (NES: 2.31, *p* < 0.001) as an adaptation to cellular oxidative stress may trigger an inflammatory cascade and protein translation driving the improper processing of proteins with overwhelming accumulation of misfolded proteins increasing stress response (unfolded protein response, NES: 2.2, *p* < 0.001). In keeping with the observed cell cycle arrest and the importance of checkpoints for the regulation of DNA damage pathways, the top two negatively enriched pathways elicit control on the cell cycle: mitotic spindle preparation (NES: −2.43, *p* < 0.001) and negative enrichment of the G2/M cell cycle checkpoint (NES: −1.79, *p* < 0.001).

Importantly, when DEGs were extracted, regardless of fold change or significance, and grouped based on the available gene sets, an overall *Z* score of differences in the expression by comparison with the mean was calculated and the statistical significance between conditions determined using Kruskal–Wallis one‐way analysis of variance (ANOVA) testing (Figure 6C). KDM4i and PARPi monotherapy showed reduction in the ubiquitin response consistently, suggesting an inability to recruit necessary factors to a site of damage to initiate repair. This reduction was further enhanced with the combination treatment.

As may be expected with small molecule targeting of an epigenetic regulator, significant changes were observed in chromatin remodelling with the KDM4i monotherapy, comparable to olaparib alone, and with the combination treatment, this was decreased further highlighting the interaction between the two agents to further dampen re‐modelling of the chromatin that would allow access for the recruitment of damage proteins. When individual DNA damage pathways were considered KDM4i alone had a modest effect, but importantly, non‐homologous end joining (NHEJ) and alt‐NHEJ were significantly reduced by the combination treatment highlighting the potential way KDM4i sensitises cells to the loss of homologous recombination with PARPi (Figure 6C; Figure S6).

## DISCUSSION

Aberrant methylation patterns of fundamental oncogenes and tumour suppressors alongside changes to histone marks controlling chromatin availability and remodelling are recognised in AML and known to be responsible for leukaemic transformation.

Previously, it has been shown that *KDM4A* depletion in specific diseases causes cell death by interfering with 53BP1 recruitment to sites of damage,^31^ a fundamental step in repair. In contrast, we have shown cell cycle arrest and increased p53‐independent cell death in response to KDM4Ai in p53 null cell lines. In solid isocitrate dehydrogenase (*IDH*)‐mutated tumours, disruption of KDM4A demethylase activity subsequently accumulated oncometabolites,^32^ such as 2‐hydroxyglutarate (2‐HG) or αKG, that suppress DNA repair and disrupt chromatin signalling.^33^, ^34^ In AML, our data confirm the role of KDM4 in the control of DNA damage and chromatin signalling, highlighting the potential for therapeutic targeting and the role of oncometabolites as a mechanism of KDM4i action.

High expression of PARP has been shown to predict poor survival^35^, ^36^ suggesting the potential of PARP inhibitors as therapy in AML.^37^ In *MLL‐AF9*‐driven leukaemia, the responsiveness of cells to PARP inhibitors has been linked to HOXA9 depletion enhancing their toxicity.^38^ Loss of HOXA9 following *KDM4A* depletion previously shown upon shRNA KD of KDM4A^8^ identifies the potential of a combination therapy with PARPi. Indeed, KDM4A monotherapy showed potential independent of subtype of AML, and in our *MLL‐AF9* leukaemia model, its anti‐leukaemic effect could be enhanced with addition of PARPi, olaparib or talazoparib which have shown efficacy in solid tumours.^39^ Opposed to this, previous studies have shown *MLL* rearranged AML to be fundamentally resistant to PARP inhibition due to the increased expression of compensatory homologous recombination proteins.^40^ Our data show in combination with KDM4i the double strand breaks induced by PARPi were increased, and this translated into more effective cell death. A reduction in NHEJ with KDM4i and PARPi combination suggests a switch in AML cell reliance to alternative repair pathways following the loss of KDM4A and subsequent reduction in homologous recombination, thus a route of exploitation for combination treatment.

It has been observed that following the initiation of DNA damage and the accumulation of 𝛾H2AX, RNF8/RNF168‐mediated KDM4A degradation at the protein level, without mRNA alteration, resulted in the removal of KDM4A from chromatin and a decreased stability dependent on ATM activation.^31^ Hypothetically, such loss of KDM4A protein following the recognition of PARPi‐induced DNA damage infers that KDM4i plus PARPi combination exacerbates further the direct inhibition of KDM4A, with the potential of a feedback mechanism to control the levels of expression. Importantly, PARP retention potential may account for the variance in the potency of the different inhibitors.

We have shown the potential of combination strategies involving epigenetic regulator, KDM4A and DNA and protein modifier, PARP in varying AML subtypes for the first time. Combination treatment elicited an increased cell death and attenuated proliferation with a cell cycle arrest, effects that persisted on drug removal from the culture. Monotherapy inhibition of homologous recombination through KDM4i caused an increased reliance of AML cells on alternative repair pathways sensitising cells to PARPi with an accumulation of damage leading to selective leukaemia cell death. These results warrant future preclinical evaluation of this combination strategy in AML and possibly other KDM4A high cancers.

## AUTHOR CONTRIBUTIONS

Conceptualisation: XH and HJ; methodology: LM, RPB, PAP, TP, HW; validation: LM; formal analysis: LM, TP, JB‐S; investigation: LM, XH; resources: XH; data curation: LM, JB‐S, HW, HGJ, XH; writing—original draft preparation: LM, HGJ; writing—review and editing: LM, HGJ, XH; supervision: XH, HGJ; project administration: LM; funding acquisition: XH. All authors have read and agreed to the published version of the manuscript.

## FUNDING INFORMATION

This research was funded by Adam Renwick Martin‐Friends of Paul O'Gorman Ph.D. Studentship.

## CONFLICT OF INTEREST STATEMENT

The authors declare no conflict of interest.

## INFORMED CONSENT STATEMENT

Primary human AML samples were from Paul O'Gorman Leukaemia Research Centre's haematological cell research biobank (with approval of the West of Scotland Research Ethics Committee 4). Their use was authorised following project review by the Research Tissue Biobank's scientific subcommittee, and with the informed consent of donors.

## Supporting information


Figure S1.

Figure S2.

Figure S3.

Figure S4.

Figure S5.

Figure S6.

Data S1.

## Data Availability

Data files are available in the Gene Expression Omnibus (GEO): GSE125376 and GSE164437.
