# Supplementary figures and images for: Selective small molecule targeting of KDM4 as a therapeutic strategy to reduce proliferation of acute myeloid leukaemia
Source: Br J Haematol. 2026 Feb 1;208(4):1240–51. doi: 10.1111/bjh.70351 (PMC13071495; doi:10.1111/bjh.70351)

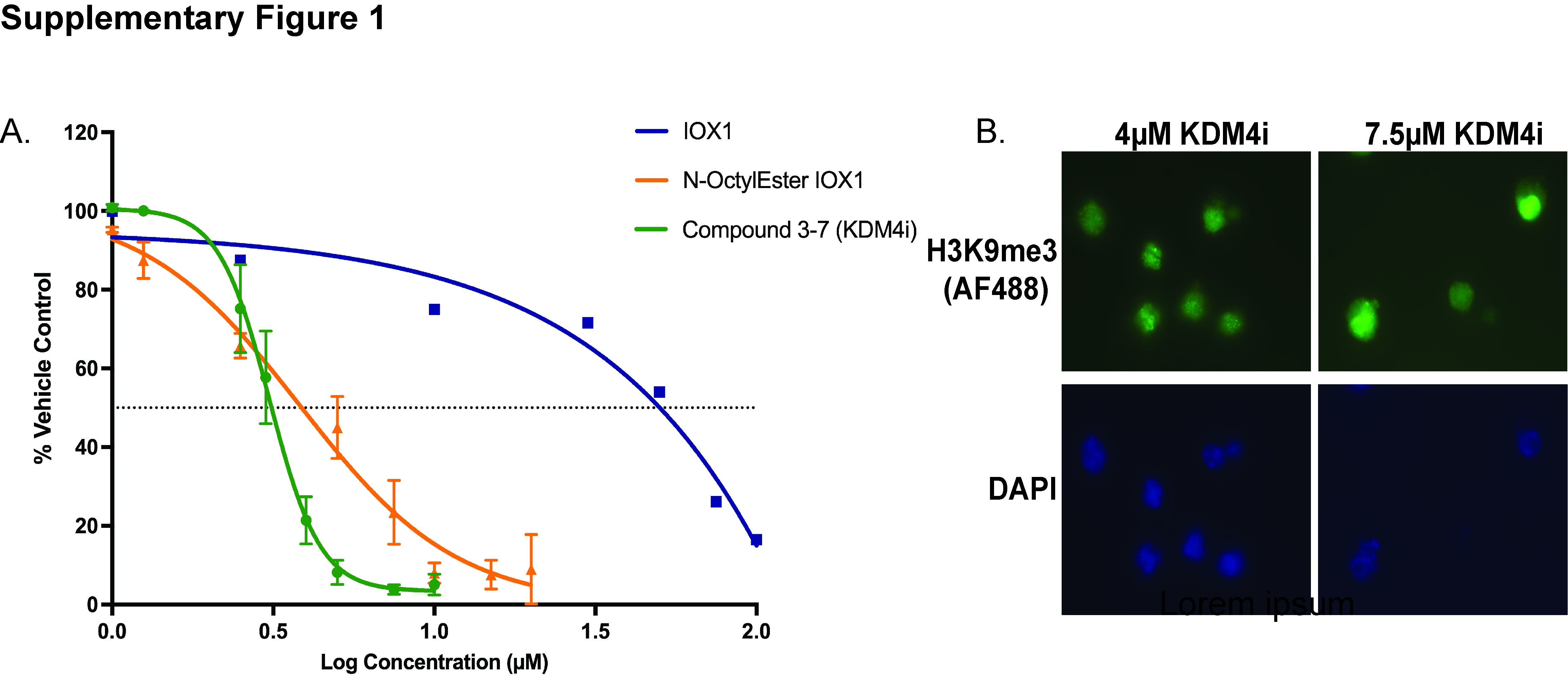

Supplement: Supplementary file 1 — Figure S1. [file BJH-208-1240-s001.tif]

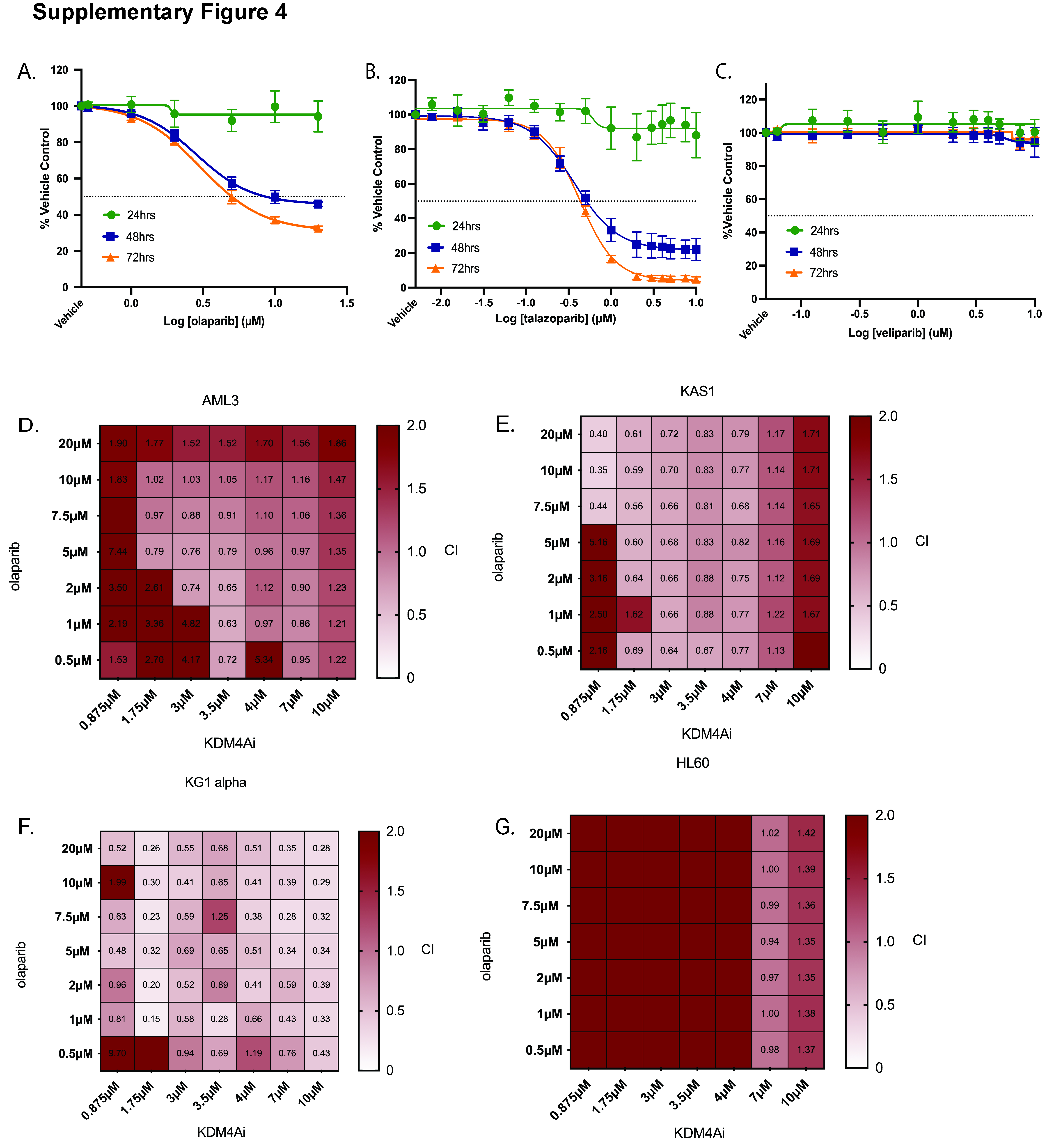

Supplement: Supplementary file 4 — Figure S4. [file BJH-208-1240-s007.tif]

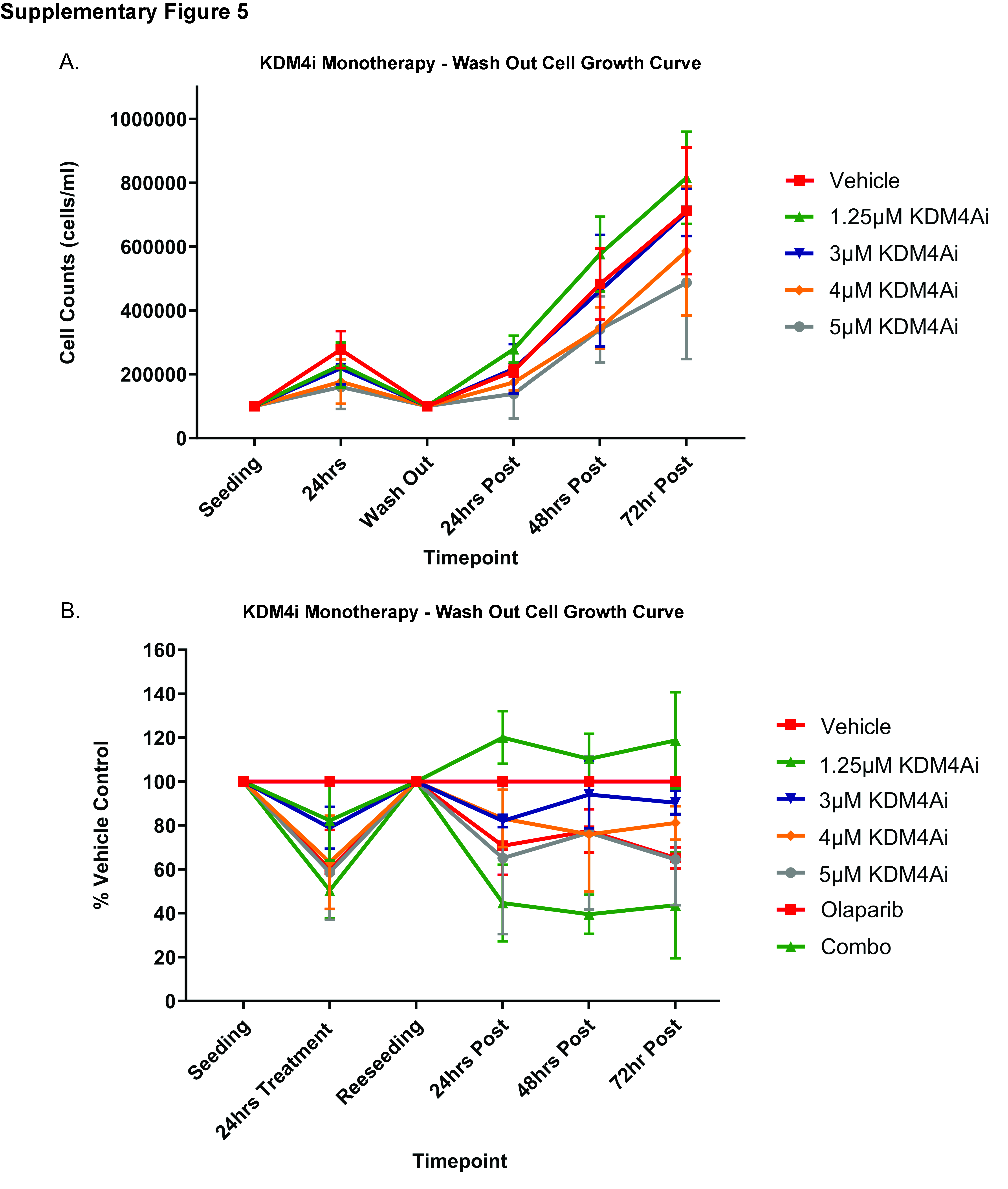

Supplement: Supplementary file 5 — Figure S5. [file BJH-208-1240-s004.tif]

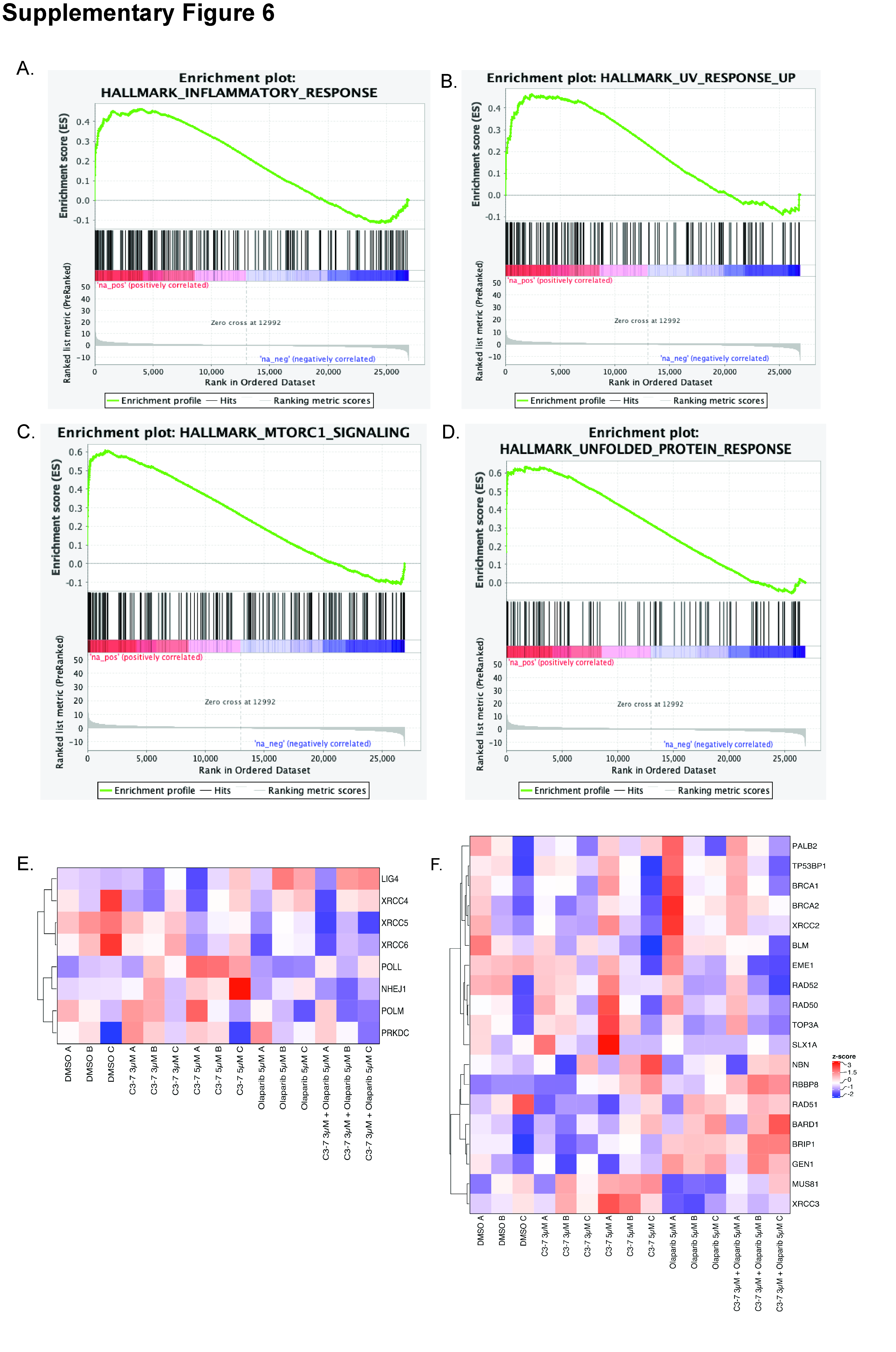

Supplement: Supplementary file 6 — Figure S6. [file BJH-208-1240-s003.tif]
