# Supplementary material for: Selective small molecule targeting of KDM4 as a therapeutic strategy to reduce proliferation of acute myeloid leukaemia
Source: Br J Haematol. 2026 Feb 1;208(4):1240–51. doi: 10.1111/bjh.70351 (PMC13071495; doi:10.1111/bjh.70351)
Supplement: Supplementary file 7 — Data S1. [file BJH-208-1240-s006.docx]

Supplementary Materials and Methods

**Cell Culture**

All AML suspension cell lines were maintained in Roswell Park Memorial Institute Medium (RPMI) supplemented with 10/20% (v/v) FBS (Sigma Aldrich) and 1% (v/v) L-Glutamine (Invitrogen) at 37°C with 5% CO_2_.

**Trypan Blue Dye Exclusion**

Cell viability of suspension cell lines and primary samples were routinely checked for cell maintenance using a haemocytometer. Trypan Blue (sigma) was prepared from powder at 0.4% (w/v) in PBS and filtered through a 0.4mM syringe filter with a 5ml syringe.

**Resazurin**

Cells were seeded in a 96 well format and treated with drug for varying lengths of time. At endpoint, 50nM final concentration resazurin was added to cell cultures. After 4hrs of incubation at 37^o^c the fluorescence of the plate was read at 535ex and 590em and normalised to blank and vehicle control wells.

**Cytospin**

Shandon Cytoclips, filter cards, sample chambers and glass slides were assembled as per manual instructions. Following drug treatment, approximately 10,000 cells were collected, washed in PBS, and re-suspended in 100-200μL media before being centrifuged at 400g for 5 minutes onto the labelled glass slides. The slides were air-dried overnight before being fixed in 100% Methanol for 7minutes. The slides were washed and left to dry before being stained with neutral dyes, May Grünwald (a mix of acidic eosin stain and basic methylene blue) and Giemsa stain (a mix of acidic eosin stain and basic azure of methylene) to visualise the nuclei and cytoplasm.

**Flow Cytometry**

Annexin V/Dapi staining of cells was carried out as described(40). For cell cycle, cells were fixed using 70% EtOH and stored overnight at -20°C overnight or for a maximum of one week then stained with a PI (1mg/mL) / Ribonuclease A (5mg/mL) solution, incubated for 15mins at 37°C then analysed immediately. CD33,CD86 staining was carried out for 30mins at 4°C, before DAPI was added and live cells analysed (CD33; BV510 Clone HIM-3-4, BD Biosciences) (CD86; PerCP Cy5.5, Clone FUN-1, BD Biosciences). Flow Cytometry analysis was carried out using FACS Canto II flow cytometer (BD Biosciences). Data was acquired using BD FACS Diva software (BD Biosciences) and analysed using FlowJo software (Tree Star Inc, Ashland, USA).

**Immunofluorescence**

Immunofluorescence was carried out using Hendley-Essex 12 well glass microscope slides coated with Poly-L-Lysine. Cells were fixed in 4% formaldehyde in PBS, permeabilized in 0.5% Triton-X-100 PBS followed by 2hrs of blocking in 5% BSA, 0.2% Triton-X-100 TBS. Primary antibody diluted 1:500 in blocking solution slides incubated overnight in a humidified chamber at 4^o^C. Following appropriate washes using PBS 0.1% Tween 20 (PBST), secondary antibody (1:500 dilution in blocking solution) was applied for 1hr at room temperature. Images were captured at 40x/100x magnification on the Zeiss Axioimager M1 Epifluorescence and Brightfield Microscope.

**Colony Forming Assay**

Colony forming unit (CFU) assays using cell lines was carried out using Methocult M3231 (Stem Cell Technologies) and Methocult H4230 as previously described(40). Following plating of cells following treatment, the mixtures were split across 3 wells and cultured at 37°C with 5% CO_2_ for 7 days prior to colony assessment using an inverted light microscope and representative image acquisition. Colonies were assessed as >50 cells clustered with clear margins to the next.

**Synergy Analysis**

Fraction affected (Fa<1) following resazurin analysis was inputted into either CompuSyn software (41) or Synergy Finder (42) developed by the University of Helsinki.

Each software outputs a score which defines synergism or antagonism, by comparing the difference in Fa between monotherapy and combination.

**Combination Index**

Mathematically CompuSyn algorithm collates concentration and Fa before plotting IC_50_ curves, and using the below equation determines a combination index (CI):

CI=D1D1x+D2D2x*CI=D1D1x+D2D2x*

If CI < 1 the combination of the two concentrations is defined as synergistic however, when the CI > 1 the combination is defined as antagonistic, with one of the drugs interfering with the action of the other.

**Bliss Independence Method**

Bliss Independence, an alternative synergy analysis tool uses a mathematical approach to assess the significance of the combination effect based on probability of the individual effects of the corresponding monotherapies resulting in the observed effect of the combination, applying the equation below to each combination concentration: -

CI= Ea+ Eb−(EaEb)Eab*CI= Ea+ Eb−(EaEb)Eab*

Synergy Finder (42) provides a user-friendly interface for the application of a range of synergy equations and heatmap outputs to show areas of synergy based on changes of the effect. By comparison of each combination with monotherapy, a synergy score shows the area of synergy. Synergy scores below 0 are antagonistic, with >0 being synergistic

**RNA Sequencing analysis**

Prior to RNA sequencing library preparation samples were analysed using Agilent RNA 6000 Nano Kit. Following sequencing quality of the samples was assessed using FastQC version 0.11.5, before sequences were trimmed using TrimGalore version 0.4.4_dev. The samples were normalised to the reference genome GRCh38 using hisat2 and a count matrix generated using featureCounts version 1.6.1 before PCA plot and identification of differentially expressed genes (DEG) was carried out using DESeq2. The genes were annotated using the R package biomaRt version 2.34.2. Differential expression pathway analysis was carried out using Reactome PA in R with log-2-fold change of 1.5 as a threshold, alternatively using FGSEA package in R, a rank taking both p-value and log2 fold change was considered.

**Wash-out Study**

Monotherapy and combination doses of KDM4i and PARPi olaparib were applied to THP-1 cells in a six well format for 24hrs, following this incubation cells were pelleted and washed with PBS before reseeding to 1x10^5^/mL and their growth monitored for 72 hr. by trypan blue cell exclusion and the average of three technical counts taken

**Trypan Blue Dye Exclusion**

Cell viability of suspension cell lines and primary samples were routinely checked for cell maintenance using a haemocytometer. Trypan Blue (sigma) was prepared from powder at 0.4% (w/v) in PBS and filtered through a 0.4mM syringe filter with a 5ml syringe.

**Resazurin**

Cells were seeded in a 96 well format and treated with drug for varying lengths of time. At endpoint, 50nM final concentration resazurin was added to cell cultures. After 4hrs of incubation at 37^o^c the fluorescence of the plate was read at 535ex and 590em and normalised to blank and vehicle control wells.

**Cytospin**

Shandon Cytoclips, filter cards, sample chambers and glass slides were assembled as per manual instructions. Following drug treatment, approximately 10,000 cells were collected, washed in PBS, and re-suspended in 100-200μL media before being centrifuged at 400g for 5 minutes onto the labelled glass slides. The slides were air-dried overnight before being fixed in 100% Methanol for 7minutes. The slides were washed and left to dry before being stained with neutral dyes, May Grünwald (a mix of acidic eosin stain and basic methylene blue) and Giemsa stain (a mix of acidic eosin stain and basic azure of methylene) to visualise the nuclei and cytoplasm.

**Flow Cytometry**

Annexin V/Dapi staining of cells was carried out as described(40). For cell cycle, cells were fixed using 70% EtOH and stored overnight at -20°C overnight or for a maximum of one week then stained with a PI (1ug/mL) / Ribonuclease A (5mg/mL) solution, incubated for 15mins at 37°C then analysed immediately. Flow Cytometry analysis was carried out using FACS Canto II flow cytometer (BD Biosciences). Data was acquired using BD FACS Diva software (BD Biosciences) and analysed using FlowJo software (Tree Star Inc, Ashland, USA).

**Immunofluorescence**

Immunofluorescence was carried out using Hendley-Essex 12 well glass microscope slides coated with Poly-L-Lysine. Cells were fixed in 4% formaldehyde in PBS, permeabilized in 0.5% Triton-X-100 PBS followed by 2hrs of blocking in 5% BSA, 0.2% Triton-X-100 TBS. Primary antibody diluted 1:500 in blocking solution slides incubated overnight in a humidified chamber at 4^o^C. Following appropriate washes using PBS 0.1% Tween 20 (PBST), secondary antibody (1:500 dilution in blocking solution) was applied for 1hr at room temperature. Images were captured at 40x/100x magnification on the Zeiss Axioimager M1 Epifluorescence and Brightfield Microscope.

**Colony Forming Assay**

Colony forming unit (CFU) assays using cell lines was carried out using Methocult M3231 (Stem Cell Technologies) and Methocult H4230 as previously described(40). Following plating of cells following treatment, the mixtures were split across 3 wells and cultured at 37°C with 5% CO_2_ for 7 days prior to colony assessment using an inverted light microscope and representative image acquisition.

**Wash-out Study**

Monotherapy and combination doses of KDM4i and PARPi olaparib were applied to THP-1 cells in a six well format for 24hrs, following this incubation cells were pelleted and washed with PBS before reseeding to 1x10^5^/mL and their growth monitored for 72 hr. by trypan blue cell exclusion and the average of three technical counts taken

Supplementary Figure 1: - Potency and targeting of KDM4A of Compound 3-7 in THP-1 cells.

*(A.) THP-1 cells treated for 48hrs with existing KDM inhibitors IOX-1(blue) and n-Octyl Ester derivative of IOX1(orange) alongside novel KDM4i (Compound 3-7)(green) in monotherapy. IC50 was determined by 4hr incubation with resazurin, and percentage change calculated relevant to the vehicle. (n=3) (B.) THP-1 cells were treated for 72 hr with compound 3-7, the cells were then fixed and stained for H3K9me3 (Alexa Fluor 488 – Green) and imaged using the AxioVision M1 fluorescence microscope. DAPI was used as a nuclear counterstain Images taken at 100x magnification. Representative images of additional two concentrations quantified in Figure 2; n=1 biological replicate with reproducible results confirmed by multiple fixations.*

Supplementary Figure 2: - Confirmation of phenotype in Mv:4-11 cell

*(A.) Varying concentrations around the IC50 of compound 3-7 was applied to Mv:4-11 cells and the metabolism of resazurin measured at different timepoint green 24hrs, blue 48hrs, orange 72hrs treatment, normalised to the vehicle control, and analysed by non-linear regression to calculate an EC50 value (B.) Apoptosis phenotype assessed by Annexin V/ Dapi flow cytometry and % of cells in each stage of apoptosis cycle n=3 (biological replicates). Stages of apoptosis are gated as a percentage of total cell, with (C.) representative dot plot images. Following monotherapy combination inhibition effect was determined using resazurin and the combination index (CI) calculated by CompuSyn.* *CI<1 is synergistic, CI~1 is additive and CI>1 is antagonistic. Heat map of representative CI values were generated using GraphPad. (n=3) with combinations involving PARPi with varying PARP trapping potential (D.) olaparib (E.) talazoparib (F.) veliparib. Combination analysis of the same inhibition data was carried out using the Bliss Independence method using Synergy Finder and confirmed the synergistic effect of the combination treatment (G.) olaparib (H.) talazoparib (I.) veliparib. The synergy scores are represented on a heat map with hotspots of synergy (scores below -10) represented by red colouring, and spots of antagonism (above 10) in green, white areas (-10 to 10) represent the two drugs being additive at those concentrations. (J.) Colony forming potential of cells following treatment for 24 or 48hrs before drug was removed and cells resuspended in semi-solid media. Colony numbers per 1000 plated for each condition after treatment for 24/48hrs. respectively.*

Supplementary Figure 3:- Extended GO term analysis of Figure 2*D/E*

*RPA of 276 genes common to all three-treatments identified (D.) All significantly upregulated pathways ordered by significance, including metabolic process and transcriptional signalling. (E.) All significantly downregulated pathways ordered by significance, including metabolic processes, cell cycle and chromatin organisation.*

Supplementary Figure 4: - Monotherapy of PARPi show varying effects in THP-1 cells with varying combination effects in cells representing different AML subtypes.

*Varying concentrations around the IC50 of compound varying PARPi was applied to THP-1 cells and the metabolism of resazurin measured at different timepoints, 24,48 or 72hrs treatment, normalised to the vehicle control, and analysed by non-linear regression to calculate an EC50 value (A.) olaparib (B.) talazoparib (C.) veliparib. (D-F) Cell’s line which exhibited different monotherapy responses were treated in combination with KDM4i and olaparib for 48 hr using resazurin assay as a readout of cellular metabolism. Fraction affected as a percentage of vehicle control was analysed by CompuSyn analysis to output a CI value. These CI values were plotted in a heatmap matrix using GraphPad Prism software with gradient applied to show effect, values > 2 have a generic colour applied, missing values were >10, n=3, 3 biological replicates each as a mean of technical triplicates. (D.) OCI-AML3 (E.) Kasumi-1 (F.) KG-1𝛼 (G.)HL60, blank squares represent values above range with no synergy.*

Supplementary Figure 5: - Removal of KDM4i in monotherapy allows recovery at lower concentrations in THP-1 cells.

*Wash out of KDM4i monotherapy at higher concentrations. Delayed growth of cells with 24 hr treatment followed by 72 hr observation. Cells were treated for 24 hr with compound 3-7 and olaparib alone or in combination before the drug was washed out and the cell growth monitored for a further 72 hr by trypan blue cell counts shown as (A.) cells/mL or (B.) %Vehicle control. Overall growth of cells for observation period, dots show mean ± SEM, n=3 3 biological replicates each as a mean of technical duplicates.*

Supplementary Figure 6: - Significant changes in gene set enrichment associated with cellular stress signalling pathways following KDM4 inhibition in THP-1 cells.

*GSEA of differentially expressed genes identifies an upregulation of cellular responses to stress in the combination treatment when the effect of the monotherapies has been removed to leave only synergistic genes altered in the combination (A.)Inflammatory Response (B.) UV Response (C.) mTORc Signalling (D.) Unfolded protein response.*
